# Seasonal Affective Disorder and the Microbiota–Gut–Brain Axis: Circadian Disruption, Tryptophan Metabolism, and Psychobiotic Potential of *Lacticaseibacillus rhamnosus* GG

**DOI:** 10.3390/nu18142364

**Published:** 2026-07-18

**Authors:** He Liu, Xin Kuang, Xinyan Zheng

**Affiliations:** School of Exercise and Health, Shanghai University of Sport, Shanghai 200438, China; 2421526015@sus.edu.cn (H.L.); 2421518038@sus.edu.cn (X.K.)

**Keywords:** seasonal affective disorder, *Lacticaseibacillus rhamnosus* GG, microbiota–gut–brain axis

## Abstract

Seasonal affective disorder (SAD) is a recurrent mood disorder associated with reduced photoperiod exposure and circadian disruption during autumn and winter. Emerging evidence links SAD to alterations in serotonergic signaling, neuroimmune activity, metabolism, and the microbiota–gut–brain axis; however, the causal relationships among these systems remain incompletely understood. A structured search of PubMed, Web of Science, and Scopus identified relevant publications from 2000 to 2025, with clinical and preclinical evidence evaluated separately. Proposed links between circadian misalignment, inflammatory signaling, and tryptophan metabolism toward the kynurenine pathway are based largely on associative and preclinical findings rather than confirmed mechanisms in SAD. The microbiota–gut–brain axis in SAD is likely bidirectional, as seasonal changes in feeding behavior, physical activity, and circadian phase may themselves influence gut microbial composition and function. Accordingly, microbiome alterations in affective disorders may reflect both potential upstream modulators and downstream consequences of disease-related behavior. Psychobiotics have been proposed as modulators of gut–brain communication in affective disorders. Among candidate strains, *Lacticaseibacillus rhamnosus* GG (formerly *Lactobacillus rhamnosus* GG; LGG) has shown effects on intestinal barrier function, immune signaling, and host tryptophan metabolism in preclinical studies. However, evidence derives largely from animal or non-seasonal depression models, and direct evidence in SAD is lacking. Thus, LGG should be considered a mechanistically plausible candidate for future investigation rather than an established therapy. This review synthesizes evidence on circadian regulation, serotonergic and tryptophan metabolism, and microbiota–gut–brain interactions in SAD, and highlights mechanistic gaps for future studies.

## 1. Introduction

Seasonal affective disorder (SAD) is a recurrent subtype of major depressive disorder characterized by depressive episodes that typically occur during autumn and winter and remit during spring or summer [1,2]. In addition to emotional symptoms, patients with SAD frequently exhibit atypical neurovegetative features, including hypersomnia, hyperphagia, carbohydrate craving, fatigue, and weight gain, suggesting the involvement of neuroendocrine and metabolic dysregulation [3,4]. Reduced photoperiod exposure and circadian rhythm disruption are among the most consistently implicated features of SAD, whereas monoaminergic, immune and metabolic alterations have also been reported [5,6,7]. Seasonal changes in dietary behavior, energy intake, physical activity and circadian timing may be relevant to this broader phenotype [8], but their causal contribution remains uncertain because these changes may also arise as consequences of seasonal mood symptoms. Direct SAD-specific evidence linking these processes into a unified pathogenic mechanism is therefore limited.

Among the proposed neurobiological mechanisms, serotonergic dysfunction has been extensively investigated, with seasonal alterations in serotonin (5-hydroxytryptamine, 5-HT) synthesis, transporter binding, and receptor-related activity reported across human and experimental studies [9,10,11]. Tryptophan metabolism has also been implicated in affective disorders, particularly through inflammatory regulation of the kynurenine pathway. Evidence from broader inflammatory, depression-related, and preclinical research links indoleamine 2,3-dioxygenase 1 (IDO1)-associated tryptophan metabolism with kynurenine pathway activity and neuroactive metabolites relevant to oxidative stress, cognition, and depressive phenotypes [12,13,14,15,16]. However, these findings do not establish a linear sequence from inflammation to kynurenine activation and serotonin deficiency in SAD.

Microbiome research has further expanded interest in the microbiota–gut–brain axis as a bidirectional communication framework linking neural, endocrine, immune, and metabolic processes [16,17]. In depressive disorders, alterations in gut microbial composition have been reported in clinical cohorts [18]. Clinical studies have also examined intestinal integrity and inflammatory markers in major depressive disorder (MDD) populations [19,20], while large population-based studies have identified associations between specific microbial taxa and depressive symptoms [21]. More recent primary studies report associations between microbial features and systemic inflammatory profiles [22] and, separately, among gut microbial composition, cytokine levels, and kynurenine-pathway metabolites [23]. These findings remain associative and do not establish that microbiota alterations drive inflammatory or tryptophan-related abnormalities in SAD. Seasonal light exposure, dietary habits, and circadian disruption may also affect microbial rhythmicity and metabolite production [24,25]. Nevertheless, direct evidence in SAD remains sparse, and directionality is unresolved: changes in mood, sleep, diet, feeding behavior, and physical activity during symptomatic periods may themselves reshape the microbiome. Microbiota-related findings should therefore not be interpreted as evidence of an established upstream cause of SAD.

Against this background, psychobiotics have been investigated as potential modulators of gut–brain communication in broader affective disorders [26]. Among candidate strains, *Lacticaseibacillus rhamnosus* GG [27] (formerly *Lactobacillus rhamnosus* GG; LGG) has attracted interest because preclinical and broader microbiome studies have examined its associations with intestinal barrier, immune, and metabolic outcomes [28,29]. However, direct clinical evidence supporting LGG in SAD is lacking, and mechanistic arguments are derived largely from animal studies, non-seasonal depression models, or broader affective-disorder research [16,26,30,31]. These findings provide a rationale for future investigation rather than evidence that LGG modifies SAD pathophysiology or has established therapeutic efficacy. Any microbiota-targeted approach should therefore be considered investigational and potentially adjunctive to established evidence-based SAD treatments, including bright light therapy [4]. Emerging precision-psychobiotic research further suggests that responses may vary with baseline microbial composition, dietary patterns, circadian status, and host metabolic phenotype [32].

Accordingly, this review examines SAD from the perspectives of disease characteristics, neurobiological mechanisms, and intervention, with emphasis on circadian dysregulation, serotonergic and tryptophan–kynurenine pathways, microbiota–gut–brain interactions, and nutritional regulation. It critically distinguishes direct evidence in SAD from findings derived from non-seasonal depression, broader clinical populations, and preclinical models, and evaluates the current limitations of these evidence bases. Microbiota-targeted strategies, particularly LGG, are discussed as mechanistically plausible candidates for future adjunctive investigation rather than established treatments for SAD.

## 2. Method

A structured literature search was conducted in PubMed, Web of Science, and Scopus for publications from 2000 to 2025. Search terms included combinations of “seasonal affective disorder”, “circadian rhythm”, “microbiota–gut–brain axis”, “tryptophan metabolism”, “kynurenine pathway”, “*Lacticaseibacillus rhamnosus* GG”, and “psychobiotics”.

Studies were included when they provided original experimental or clinical evidence relevant to SAD or related affective disorders, including human investigations and animal studies. Reviews and editorials were considered primarily for relevant conceptual or mechanistic context, while non-English publications were considered when they contributed pertinent evidence to the scope of the review. Preclinical and clinical evidence were evaluated separately throughout the manuscript, with explicit distinction between findings derived from animal models and those obtained in human populations.

Given the limited availability of SAD-specific mechanistic studies, evidence from related depressive or stress-related conditions and experimental models was also considered where relevant. Such evidence was interpreted cautiously and was not treated as equivalent to direct evidence in SAD, particularly when assessing causal mechanisms, translational relevance, or therapeutic implications.

## 3. Seasonal Affective Disorder

### 3.1. Definition, Clinical Characteristics, and Seasonal Neurobiology

SAD is a subtype of mood disorder characterized by recurrent depressive, manic, or hypomanic episodes occurring in specific seasons. The most common form, winter-pattern SAD, typically emerges during late autumn or early winter and remits spontaneously during spring or summer [1]. In addition to depressed mood, patients frequently exhibit atypical neurovegetative symptoms including hypersomnia, hyperphagia with carbohydrate craving, fatigue, psychomotor retardation, reduced motivation, and social withdrawal [1,3]. Compared with non-seasonal depression, SAD is more explicitly characterized by a photoperiod-linked clinical course and circadian disturbance, whereas the involvement of metabolic dysregulation is supported by its atypical neurovegetative features and associated metabolic changes. These characteristics suggest that seasonal environmental factors are particularly relevant to SAD pathophysiology, although the relative contribution of individual circadian and metabolic mechanisms remains incompletely defined.

SAD exhibits a pronounced latitudinal distribution and high recurrence rate, with increased prevalence observed in regions characterized by reduced winter daylight exposure [2]. Epidemiological evidence associates reduced photoperiod and seasonal circadian disruption with mood vulnerability. Seasonal changes in sleep timing, dietary behavior, feeding rhythm, physical activity, and metabolic homeostasis have also been proposed as relevant to the broader SAD phenotype [1,33]. Broader chrononutrition research suggests that meal timing, energy intake, and nutrient metabolism can interact with circadian signaling and microbial rhythmicity [34], providing a rationale for investigating similar relationships in SAD rather than establishing a disorder-specific pathway.

Altered photic signaling and circadian rhythm dysregulation represent some of the most consistently implicated biological features of SAD. Light information is detected by intrinsically photosensitive retinal ganglion cells (ipRGCs), which are particularly responsive to blue light and project directly to the suprachiasmatic nucleus (SCN), the master circadian pacemaker. Through regulation of melatonin secretion and circadian phase alignment, the SCN coordinates sleep–wake cycles, endocrine rhythms, metabolism, and emotional regulation. Clinical evidence indicates that patients with SAD may exhibit reduced ipRGC responsiveness during winter, including diminished post-illumination pupil response following blue-light stimulation, suggesting impaired biological sensitivity to seasonal light exposure [3].

Based on these observations, the phase-delay hypothesis proposes that circadian rhythms in SAD are delayed relative to the external light–dark cycle and sleep schedule. Morning bright light therapy is clinically effective in SAD, and its therapeutic effects are commonly attributed, at least in part, to circadian phase advancement and resynchronization [4]. Beyond these relatively well-established circadian associations, broader circadian and experimental research links circadian misalignment with metabolic, oxidative, inflammatory, and energy-homeostatic disturbances [25,35]. However, whether these processes directly contribute to neurovegetative symptoms in SAD remains uncertain.

Beyond circadian abnormalities, SAD has also been associated with dysregulation of monoaminergic neurotransmitter systems, including 5-HT, norepinephrine (NA), and dopamine (DA), which are involved in mood regulation, reward processing, and motivational behavior [5]. Seasonal alterations in serotonin transporter activity and related serotonergic processes have been reported across clinical and experimental studies [6]. However, the proposed link between serotonergic abnormalities and inflammation-driven changes in peripheral tryptophan metabolism is supported mainly by broader depression, inflammatory and experimental research [36], rather than by a validated causal pathway in SAD.

Direct evidence linking gut microbial alterations to SAD remains limited. Broader chronobiological and experimental research associates photoperiod and feeding changes with microbial rhythmicity, intestinal permeability and microbial metabolite production [24,25], but directionality remains unresolved. Changes in mood, sleep, diet, feeding rhythm, and physical activity accompanying SAD may themselves reshape the microbiome; accordingly, microbiota-related changes should be regarded as part of a bidirectional, hypothesis-generating framework rather than an established upstream pathogenic mechanism.

Compared with non-seasonal depression, SAD has a more clearly defined environmental trigger and a stronger photoperiod-dependent clinical course. Its recurrent winter onset, spontaneous remission during spring or summer, and characteristic atypical symptoms suggest that seasonal biological adaptation may contribute to the disorder [37]. Hypersomnia, hyperphagia, carbohydrate craving, and weight gain are also consistent with altered energy balance and appetite regulation in the SAD phenotype [37,38]. Although SAD and major depressive disorder share several candidate mechanisms, including serotonergic, inflammatory, and hypothalamic-pituitary-adrenal (HPA) axis abnormalities, SAD is more directly characterized by photoperiod sensitivity and circadian phase alignment, with seasonal eating behavior and microbial rhythmicity representing additional proposed factors [24,25,37]. Findings derived from chronic stress or non-seasonal depression models should be extrapolated cautiously unless seasonal or photoperiod-related factors are considered.

### 3.2. Circadian and Metabolic Characteristics of SAD

Among the proposed pathogenic mechanisms of SAD, circadian rhythm disruption remains one of the most extensively investigated biological frameworks. Reduced winter photoperiod and delayed light exposure may impair alignment between endogenous circadian rhythms and the external environment, with consequences for sleep timing and circadian regulation [4]. However, direct evidence that such misalignment drives broader endocrine, neuroimmune, and metabolic abnormalities in SAD remains limited, as much of the supporting evidence derives from general circadian research, non-seasonal mood disorders, or experimental models.

Across broader circadian research, circadian disruption has been associated with HPA axis and inflammatory alterations. In a controlled human laboratory study, chronic circadian misalignment altered cortisol levels and increased several circulating inflammatory proteins [39]. These findings support biological plausibility but do not establish direct mediation of neurovegetative or affective symptoms in human SAD.

Broader circadian research indicates that gut microbial composition and function can exhibit diurnal variation [25]. Experimental studies outside SAD further show that feeding timing can alter microbial rhythmicity and associated metabolic or inflammatory patterns [40,41]. In SAD, direct evidence for disrupted microbial rhythmicity or a causal role in seasonal mood symptoms remains limited, and reverse causation is plausible because changes in sleep, dietary behavior, feeding timing, and physical activity may themselves reshape the microbiome. Outside SAD, intestinal barrier-related and endotoxin-associated inflammatory processes have also been investigated in broader neuropsychiatric contexts [20]. In a longitudinal human cohort, higher endotoxemia markers predicted greater subsequent depressive symptoms when inflammatory burden was elevated [42]. Nevertheless, these findings do not establish a sequential pathway from circadian disruption through gut barrier dysfunction and inflammation to IDO1 activation and serotonergic abnormalities in SAD.

The atypical neurovegetative profile of winter-pattern SAD suggests that altered energy balance and metabolic adaptation may be relevant components of the disorder. During winter, reduced light exposure, decreased outdoor activity, prolonged sleep duration, and altered eating behavior may be associated with lower energy expenditure and greater preference for carbohydrate-rich foods [37,38]. Broader metabolic research raises the possibility that persistent hyperphagia and winter weight gain may contribute to metabolic inflammation, insulin resistance, and further dysregulation of mood-related neuroendocrine pathways [38,43]. In this context, seasonal metabolic adaptation may therefore be conceptualized as both a consequence and a contributor to SAD pathophysiology.

Carbohydrate intake may transiently increase central tryptophan availability and serotonin synthesis by altering the ratio of tryptophan to competing large neutral amino acids [44,45]. Broader metabolic research associates energy-dense dietary patterns with increased metabolic burden and low-grade inflammation [43,46]. Microbiota- and barrier-related changes have also been reported in non-SAD metabolic and experimental contexts [46], but their relevance to seasonal mood symptoms remains uncertain.

Collectively, seasonal circadian disturbance is the best-supported component of this framework, whereas neuroimmune, metabolic, and microbiota-associated pathways remain supported to varying degrees and have not been integrated into a validated causal network in SAD.

## 4. Neurobiological Mechanisms Underlying SAD

### 4.1. Serotonergic Dysfunction and Tryptophan–Kynurenine Metabolism

5-HT is synthesized from the essential amino acid tryptophan through the rate-limiting enzyme tryptophan hydroxylase (TPH) and plays a central role in the regulation of mood, sleep, appetite, cognition, reward processing, and stress responsiveness [47,48]. Approximately 90–95% of serotonin is synthesized in enterochromaffin cells within the gastrointestinal tract, whereas the remaining fraction is produced by serotonergic neurons in the brainstem raphe nuclei [46]. Because peripheral serotonin does not readily cross the blood–brain barrier, central serotonin synthesis depends on available tryptophan; peripheral tryptophan availability and metabolism can therefore influence, but do not alone determine, central serotonergic activity [49,50]. In SAD specifically, however, direct evidence linking peripheral tryptophan–kynurenine metabolism to central serotonergic dysfunction remains limited, and much of the proposed mechanistic framework derives from broader depression, inflammatory, and preclinical research.

Tryptophan metabolism primarily proceeds through two major pathways: the serotonin synthesis pathway mediated by TPH and the kynurenine pathway mediated by IDO1 or tryptophan 2,3-dioxygenase (TDO2) [51,52]. Under physiological conditions, only a small proportion of tryptophan is utilized for serotonin synthesis, whereas approximately 90% is metabolized through the kynurenine pathway [53]. Broader inflammatory and stress-related research indicates that inflammatory signaling and HPA axis activation can alter IDO1- and TDO2-related tryptophan metabolism [36,53]. These findings support the hypothesis that serotonin and kynurenine pathway utilization may vary under specific inflammatory or stress-related conditions, rather than establishing a uniform metabolic shift in SAD.

Kynurenine metabolism generates multiple neuroactive metabolites with distinct biological functions. Quinolinic acid and 3-hydroxykynurenine exhibit neurotoxic and pro-oxidative properties, whereas kynurenic acid exerts neuroprotective effects through modulation of glutamatergic neurotransmission [17,36]. Across broader neuropsychiatric and metabolic research, kynurenine pathway dysregulation has been associated with oxidative stress, mitochondrial impairment, metabolic abnormalities, and affective disorders [35]. Depression-related and experimental studies also link altered kynurenine metabolism to changes in synaptic plasticity and neuronal resilience [54]. Whether these processes contribute directly to emotional or cognitive symptoms in SAD remains unresolved.

Outside SAD-specific studies, inflammatory signaling has been proposed as one route through which immune activation can alter tryptophan metabolism. Pro-inflammatory cytokines, including interleukin-6 (IL-6), tumor necrosis factor-α (TNF-α), and interferon-γ (IFN-γ), can regulate IDO1-related metabolism, whereas HPA axis and glucocorticoid signaling can influence TDO2 activity [53]. These mechanisms provide a biological rationale for altered serotonin precursor availability and kynurenine metabolite profiles but do not establish a causal sequence from inflammation to serotonin deficiency in SAD. Broader depression-related and experimental research also links altered kynurenine metabolite profiles with neurobiological changes relevant to mood disorders [36,54].

Evidence specific to SAD suggests seasonal alterations in serotonergic function. Neuroimaging studies have reported seasonal changes in serotonin transporter binding and serotonergic neurotransmission in SAD [31]. Broader circadian and seasonal research has associated photoperiod variation with changes in stress, inflammatory, feeding and metabolic processes [24,25], but whether these factors causally mediate seasonal serotonergic alterations in SAD remains uncertain.

Taken together, direct evidence in SAD most clearly supports seasonal alterations in serotonergic function, whereas proposed links among inflammation, HPA axis activation, IDO1/TDO2 regulation, kynurenine metabolism, and serotonin availability rely substantially on broader depression-related and preclinical evidence. These relationships should therefore be interpreted as hypothesis-generating rather than as a validated linear causal sequence in SAD, with their directionality and temporal ordering remaining unresolved.

### 4.2. Microbiota–Gut–Brain Axis in SAD

Microbiome research has substantially expanded current understanding of the microbiota–gut–brain axis in affective disorders. The microbiota–gut–brain axis represents a complex bidirectional communication network linking intestinal microbiota, gastrointestinal physiology, immune signaling, metabolic regulation, endocrine pathways, and the central nervous system [16,17]. Through neural pathways, microbial metabolites, immune mediators, and neuroendocrine signaling, gut microbiota can influence emotional behavior, cognitive function, stress responsiveness, and neuroinflammatory activity, while the brain can reciprocally regulate intestinal physiology and microbial composition through autonomic and HPA axis signaling [16,17]. In SAD specifically, however, direct evidence for microbiome alterations and their causal relationship with seasonal symptoms remains limited; much of the current mechanistic framework derives from non-seasonal depressive disorders, broader microbiome research, or experimental models.

Research across affective and stress-related neuropsychiatric conditions has increasingly examined the microbiota–gut–brain axis as a mechanistic framework [16]. Systems-level models have proposed potential interactions among gut microbial dysbiosis, intestinal barrier dysfunction, immune activation, altered microbial metabolism, and neuroendocrine dysregulation [20,55], but do not establish a unified pathogenic sequence, particularly in SAD. Clinical cohort and metagenomic studies have reported associations between depressive disorders and specific features of gut microbial composition [18,56]. Large population-based analyses have further identified associations between depressive symptoms and specific microbial taxa [21], while a recent study in treatment-naive patients with major depressive disorder reported taxonomic differences associated with a pro-inflammatory profile [22]. These findings remain associative, vary across study populations, and do not establish either directionality or SAD-specific relevance.

Caution is therefore required when extrapolating such findings to SAD, whose clinical course is characterized by predictable seasonal recurrence and strong photoperiod dependence [3,4]. Seasonal alterations in feeding behavior, carbohydrate craving, sleep timing, and energy metabolism may be relevant to microbial rhythmicity and host metabolic regulation [8,24,25]. Broader chronobiological research indicates that microbial composition and metabolic activity exhibit diurnal oscillations [25], while experimental studies outside SAD show that feeding timing can alter microbial rhythmicity and associated metabolic or inflammatory patterns [40,41]. Altered photoperiod or irregular feeding has also been associated with changes in microbial composition and metabolite production in broader experimental contexts [24]. However, whether such microbial changes mediate serotonergic, immune, or stress-related alterations in SAD remains unestablished. These findings therefore provide a rationale for investigating microbial rhythmicity in SAD rather than evidence of a causal contribution to seasonal mood vulnerability.

Intestinal barrier dysfunction has been investigated as a potential feature of depression-related pathophysiology. Clinical and experimental studies have reported associations among depressive symptoms, increased intestinal permeability, and elevated circulating endotoxin-related markers [19]. In broader human research, elevated endotoxemia markers in the context of heightened inflammatory burden have been associated with subsequent depressive symptoms [42]. Microbial-derived products such as LPS can activate inflammatory pathways including TLR4–NF-κB signaling and induce production of pro-inflammatory cytokines such as IL-1β, IL-6, and TNF-α [57]. In broader neuroimmune research, these mediators have been proposed to affect brain function through humoral signaling, peripheral immune-to-brain communication, and microglial responses [58]. However, whether intestinal barrier dysfunction and endotoxin-related inflammatory signaling contribute to neuroinflammatory alterations in SAD remains unestablished.

Microbial activity can influence host tryptophan metabolism across serotonin, kynurenine, and indole pathways [59,60,61]. In major depressive disorder, a recent multi-omics study reported associations among gut microbial composition, cytokine levels, and kynurenine-pathway metabolites [23]. In broader inflammatory and depression-related research, altered IDO1- and TDO2-associated metabolism has been linked to increased kynurenine pathway activity [35,36], but evidence that microbiota-driven inflammation causes a shift from serotonin toward kynurenine metabolism in SAD is lacking. Associations between kynurenine metabolites and broader neurobiological alterations derive largely from experimental contexts [62]. Other microbiota-derived metabolites, including short-chain fatty acids (SCFAs), secondary bile acids, and indole derivatives, have been implicated in neuroimmune and metabolic signaling [24,59]. SCFAs can modulate microglial maturation, glial metabolism, and inflammatory activity through G protein-coupled receptor signaling and histone deacetylase inhibition [63], while indole derivatives may regulate mucosal immunity and neuroinflammation pathways through aryl hydrocarbon receptor (AhR)-dependent signaling [59]. These metabolites should therefore be regarded as candidate mediators of gut–brain communication whose relevance to human SAD remains largely untested.

Preclinical studies also provide evidence for neural routes of microbiota-brain communication. Administration of *Lacticaseibacillus rhamnosus* JB-1 has been reported to alter γ-aminobutyric acid (GABA) receptor expression, suppress stress-induced corticosterone release, and improve anxiety- and depression-like behaviors in animal models through vagus nerve-dependent mechanisms [64]. These findings support vagal afferent signaling as a candidate pathway but do not establish their translational relevance to human SAD.

Collectively, the microbiota–gut–brain axis should be regarded as a bidirectional, hypothesis-generating framework in SAD rather than an established upstream pathogenic mechanism, because neither directionality nor temporal sequence has been resolved.

### 4.3. Nutritional Regulation, Chrononutrition, and Microbial Rhythmicity in SAD

Nutritional factors and meal timing may be relevant to SAD through their relationships with circadian regulation, gut microbial activity, and tryptophan metabolism. However, direct evidence that nutritional and chrononutritional factors causally contribute to SAD remains limited, and much of the mechanistic rationale derives from broader nutritional, circadian, and experimental research. Tryptophan is an essential amino acid obtained exclusively from dietary intake and serves as the primary precursor for serotonin, melatonin, and kynurenine pathway metabolites [60]. Dietary tryptophan availability can influence substrate availability for these metabolic pathways but does not alone determine central serotonergic or circadian neuroendocrine function. Patients with SAD frequently exhibit seasonal hyperphagia and carbohydrate craving during winter episodes [37], providing a rationale for investigating nutritional contributions without establishing a causal role for altered nutrient intake.

Carbohydrate-rich meals may transiently increase brain tryptophan availability by promoting insulin-mediated uptake of competing large neutral amino acids into peripheral tissues, thereby facilitating central serotonin synthesis [44]. This physiological mechanism has been proposed as one explanation for carbohydrate craving, although its compensatory role in SAD remains uncertain. Broader metabolic and dietary research associates chronic energy-dense diets with metabolic inflammation, gut microbial dysbiosis, and intestinal barrier dysfunction [46], but whether these processes exacerbate SAD symptoms remains unknown.

Broader nutritional and microbiome research indicates that dietary patterns can influence microbial tryptophan metabolism and the production of indole derivatives, kynurenine metabolites, and SCFAs involved in neuroimmune regulation [65]. In a recent crossover randomized trial in healthy adults, oral tryptophan supplementation altered duodenal microbial indole and host kynurenine pathway activity and activated aryl hydrocarbon receptor-related signaling [66]. These findings provide specific evidence that dietary tryptophan can influence intestinal tryptophan-related pathways but do not establish corresponding effects in SAD. Chrononutrition research further indicates that feeding time can influence microbial rhythmicity [67]. Experimental studies outside SAD show that time-restricted feeding or altered dietary timing can modify microbial oscillations and associated metabolic or inflammatory patterns [40,41]. Other experimental work has reported changes in microbial amino acid metabolism or inflammatory signaling under irregular feeding or altered photoperiod conditions [68], while broader circadian research has proposed additional seasonal and photoperiod-related modulation of microbial rhythmicity [24,25]. Whether these changes contribute to mood symptoms in SAD has not been established.

Chrononutrition provides a useful framework for investigating relationships between dietary timing, metabolic regulation, and SAD. In addition to the central clock located in the SCN, peripheral clocks in the liver, intestine, adipose tissue, and immune cells are responsive to feeding–fasting cycles [69]. Broader chrononutrition research links irregular or delayed meal timing with adverse metabolic outcomes [25,37]. In a controlled crossover trial in adults with overweight or obesity, late isocaloric eating increased hunger, reduced waking energy expenditure, and altered adipose metabolic pathways [70]. Related research also suggests that microbial rhythms may vary with diet composition and host circadian status [71]. In SAD, delayed circadian phase, hypersomnia, reduced daytime activity, and altered appetite could themselves change meal timing and feeding–fasting patterns, making it unclear whether chrononutritional disruption precedes seasonal symptoms or arises partly as a consequence of them.

In addition to macronutrient intake, dietary bioactive compounds may influence microbiota-related metabolic pathways. Polyphenol-rich dietary components and fermented foods have been reported to modulate gut microbial composition, intestinal barrier integrity, and tryptophan metabolism [72]. In a randomized prospective study in healthy adults, a high-fermented-food diet altered gut microbial diversity and reduced multiple inflammatory markers [73]. These findings support further investigation of microbiota-targeted dietary approaches but do not establish effects on SAD or therapeutic efficacy in seasonally affected populations.

**Figure 1:**
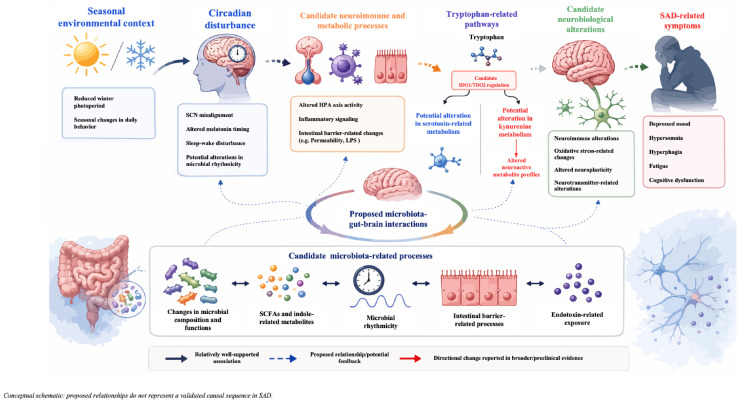
Solid dark arrows indicate relatively well-supported associations; colored dashed arrows indicate proposed relationships or potential feedback; red arrows indicate directional changes reported in broader or preclinical evidence. The figure is schematic and hypothesis-generating and does not represent a validated causal sequence in SAD. This original schematic was created by the authors using Adobe Photoshop and Microsoft PowerPoint; no published material was reproduced or adapted, and no permissions were required.

Broader nutritional psychiatry research suggests that dietary composition, nutrient availability, feeding rhythm, and baseline microbial composition may influence microbial metabolite production, host tryptophan metabolism, and responses to microbiota-targeted interventions [56,65]. Nevertheless, clinical evidence specifically evaluating chrononutrition and microbiota-targeted dietary interventions in SAD remains limited. Taken together, current evidence supports further investigation of interactions among chrononutrition, microbial rhythmicity, tryptophan metabolism, and circadian regulation in SAD, but does not establish an integrated pathogenic network or causal sequence (Figure 1).

## 5. Current Therapeutic Strategies and Psychobiotic Interventions

### 5.1. Conventional Therapeutic Strategies and Remaining Unmet Needs

Current therapeutic approaches for SAD primarily include bright light therapy, pharmacotherapy, and cognitive behavioral therapy tailored for SAD (CBT-SAD). Among these interventions, morning bright light therapy remains a first-line evidence-based treatment for SAD, with its therapeutic effects commonly attributed, at least in part, to circadian phase advancement and regulation of melatonin secretion [4,7]. It can also improve depressive and neurovegetative symptoms in SAD [4].

Pharmacological treatment commonly includes selective serotonin reuptake inhibitors (SSRIs), whose clinical effects are associated with modulation of serotonergic neurotransmission [9]. SSRIs remain widely used to reduce depressive symptom severity. CBT-SAD focuses on modifying maladaptive seasonal cognitive patterns, behavioral avoidance, and dysfunctional responses to winter-related environmental changes, thereby improving coping strategies and reducing recurrence risk [10].

Despite demonstrated clinical efficacy, conventional response is not universal. Some patients experience incomplete response, recurrence following treatment discontinuation, or treatment-related adverse effects [11,12]. Variability in clinical presentation and treatment response has therefore stimulated interest in additional adjunctive approaches. Broader affective-disorder research has implicated circadian, immune, metabolic, and microbiota-associated pathways [20], although their causal relevance and therapeutic tractability in SAD remain incompletely established. Evidence from SAD-specific observations, broader depression research, and experimental studies has likewise implicated circadian, inflammatory, metabolic, microbial, and tryptophan-related processes [24,25,35]. However, these findings do not establish an integrated causal network in SAD. They instead provide a rationale for evaluating adjunctive approaches informed by broader biological observations rather than evidence that such interventions modify a validated pathogenic network. The major therapeutic approaches currently used in SAD, together with their principal benefits, limitations, and remaining clinical needs, are summarized in Table 1.

Against this background, advances in nutritional psychiatry and microbiome research have expanded interest in microbiota-targeted interventions as potential complementary approaches in mood disorders. Seasonal variation in gut microbiota composition has been reported outside SAD [74], although reverse causation and confounding by seasonal behavior remain plausible. Broader depression and experimental research have linked microbiota-associated alterations with barrier, inflammatory, and tryptophan-related processes [20]. However, these findings do not establish a causal role in SAD. Current evidence therefore supports future evaluation of microbiota-targeted approaches as potential adjuncts to established SAD treatments rather than evidence of therapeutic efficacy.

### 5.2. Psychobiotics and Microbiota-Targeted Approaches

Psychobiotics have been investigated as potential interventions in broader affective and stress-related disorders, but direct clinical evidence in SAD remains limited. Accordingly, their relevance to SAD should currently be viewed as a mechanistic rationale for future adjunctive trials rather than evidence of established therapeutic efficacy. Psychobiotics are generally defined as probiotics investigated for potential beneficial effects on emotional behavior, stress responsiveness, or cognitive function through microbiota–gut–brain signaling pathways [26]. Proposed mechanisms involve multiple gut–brain pathways, but their empirical support varies substantially across strains, experimental models, and clinical populations [26,59].

Human mechanistic evidence in depressive disorders remains limited and heterogeneous. In one randomized add-on trial in patients with depression, a 31-day multistrain probiotic intervention was accompanied by changes in depressive symptoms, gut microbial composition, and putamen responses [75]. In a separate randomized controlled trial in patients with major depressive disorder, adjunctive *Lactobacillus plantarum* 299v reduced plasma kynurenine concentrations and improved selected cognitive outcomes, whereas IL-6, IL-1β, TNF-α, and cortisol did not significantly change [76]. A more recent mechanistic analysis of a pilot MDD trial reported selected microbiome differences but no between-group differences in inflammatory markers [77]. These findings illustrate that clinical, microbial, metabolic, and inflammatory outcomes do not change uniformly across probiotic trials and do not establish corresponding mechanisms in SAD.

Preclinical studies can provide evidence for specific candidate routes of microbiota–brain communication. For example, *Lacticaseibacillus rhamnosus* JB-1 has been reported to alter GABA receptor expression and stress-induced corticosterone responses through vagus nerve-dependent mechanisms in mice [64]. Such findings support biological plausibility but do not establish equivalent mechanisms or therapeutic effects in human SAD.

Dietary context and baseline microbial composition may contribute to variability in responses to microbiota-targeted interventions [56,65], although reliable predictors of psychobiotic response have not yet been established. Changes in mood, sleep, diet, and physical activity during treatment may themselves reshape the microbiome, complicating attribution of clinical improvement to a microbiota-driven mechanism.

Clinical evidence regarding psychobiotics in mood disorders remains heterogeneous. The contrasting findings across available trials caution against treating psychobiotics as a uniform intervention class, particularly because outcomes may depend on strain, formulation, treatment context, and study population [75,76,77,78,79]. Evidence specifically evaluating psychobiotics in SAD remains scarce, while much of the mechanistic rationale derives from chronic stress- or inflammation-based preclinical models that do not reproduce the defining photoperiod-dependent features of SAD [80,81]. Whether microbiota-targeted interventions provide clinically meaningful benefit under seasonally relevant conditions remains unknown. Current evidence supports their further evaluation as investigational adjuncts to established SAD treatments rather than as therapies with demonstrated efficacy.

### 5.3. Mechanistic Rationale for Investigating LGG in SAD

Among candidate psychobiotic strains, LGG has attracted interest because of its well-characterized probiotic properties and reported immunometabolic effects in broader experimental contexts [28,29]. However, direct clinical evidence supporting LGG in SAD is lacking, and most mechanistic arguments derive from preclinical studies, non-seasonal depression models, or broader microbiome research. Accordingly, LGG should be considered a candidate for future adjunctive investigation rather than an established therapy for SAD.

LGG is a human-derived probiotic strain originally isolated and characterized by Goldin and Gorbach in 1983, from whom the designation “GG” was derived [82]. It is a Gram-positive facultative heterofermentative bacterium characterized by high resistance to gastric acid and bile salts, mucosal adhesion capacity, and substantial stability during gastrointestinal transit [28]. LGG has consequently been widely used in probiotic foods and formulations, while its broader biological effects have been investigated across gastrointestinal, immune, and experimental neurobehavioral contexts [28,29]. These properties support its selection as a candidate strain but do not establish psychobiotic efficacy in SAD.

Experimental evidence for LGG-related barrier effects is more specific in non-SAD systems. In human intestinal enteroids and colonoids, LGG prevented IFN-γ- and irritable bowel syndrome fecal-supernatant-associated increases in epithelial permeability and loss of ZO-1 and occludin expression [83]. Broader experimental studies have also reported LGG-associated changes in barrier-related and inflammatory outcomes in disease-specific models [28,29,80]. More recently, a chronic ethanol-exposure mouse study reported attenuation of depression-like behavior and cognitive deficits together with reductions in systemic inflammatory alterations following LGG administration [15]. A chronic unpredictable mild stress (CUMS) mouse study evaluated LGG both alone and in combination with glutamine and curcumin [81], complicating attribution of effects observed under the combined regimen specifically to LGG. Collectively, these studies support candidate barrier- and immune-related effects in selected experimental settings but do not establish a corresponding causal mechanism in SAD. Moreover, these models do not reproduce the defining photoperiod-dependent features of the disorder.

Direct evidence that LGG regulates tryptophan–kynurenine metabolism in SAD is also lacking. In gnotobiotic mice, LGG has been shown to interact with dietary tryptophan-dependent metabolic processes, including increased production of the barrier-protective metabolite methylnicotinamide [84]. However, this finding does not establish that LGG acts through IDO1 or TDO2 or preserves tryptophan for serotonin synthesis in SAD. Pharmacological inhibition of IDO1 has reversed inflammation-induced depressive-like behaviors in experimental models [85]^,^ supporting the broader relevance of this pathway without demonstrating that LGG acts through IDO1 inhibition. In an LPS-exposed offspring model, LGG intervention was associated with improved gut barrier function and reduced corticosterone, alongside changes in social behavior [80]. However, whether these effects reflect a stress-axis mechanism relevant to human SAD remains untested.

**Figure 2:**
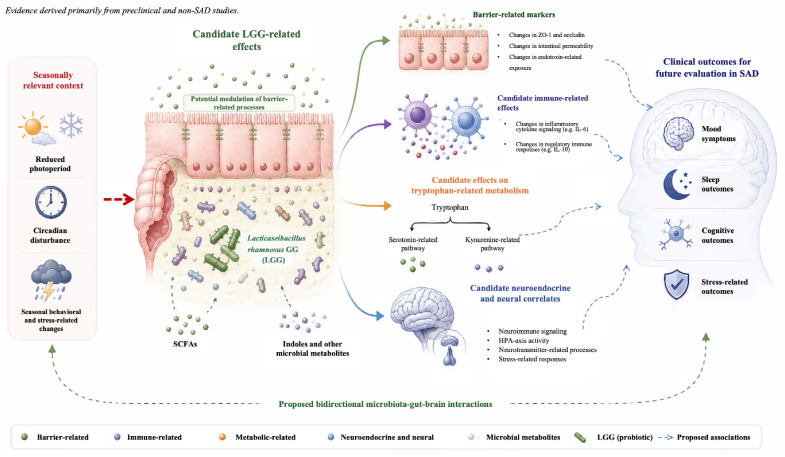
Solid colored arrows indicate candidate LGG-related effects on barrier, immune, tryptophan-related, and neuroendocrine/neural pathways; blue dashed arrows indicate proposed associations with clinical outcomes; green dashed arrows indicate proposed bidirectional microbiota–gut–brain interactions; and the red dashed arrow indicates the proposed influence of seasonally relevant conditions on the gut environment. The illustrated effects are derived primarily from preclinical, non-seasonal depression, or broader microbiome research, and their relevance to SAD remains hypothetical. Clinical efficacy of LGG in SAD has not been established. This original schematic was created by the authors using Adobe Photoshop and Microsoft PowerPoint; no published material was reproduced or adapted, and no permissions were required.

Broader psychobiotic research has proposed baseline microbial composition, dietary context, and host physiological state as potential modifiers of probiotic response [32,86], but validated predictors of LGG response in SAD have not been established. Figure 2 therefore summarizes candidate mechanisms derived primarily from preclinical and non-SAD evidence and should be interpreted as a conceptual framework rather than a validated network in SAD. Seasonal changes in mood, diet, sleep, and physical activity may themselves alter the gut microbiome and influence apparent probiotic responsiveness, complicating attribution of any observed benefit to an LGG-driven mechanism. Whether LGG provides clinically meaningful benefit in SAD remains unknown and requires evaluation in photoperiod-relevant mechanistic studies and clinical trials as a potential adjunct to established treatments.

## 6. Limitations of Current Evidence

Several limitations constrain interpretation of the current evidence linking circadian, metabolic, microbiota-related, and psychobiotic processes to SAD. A central limitation is the scarcity of direct SAD-specific evidence, as much of the proposed framework derives from non-seasonal depressive disorders, broader circadian and metabolic research, or preclinical models. These evidence levels are not interchangeable because findings from general depression or experimental settings do not demonstrate that the same relationships occur under the defining photoperiod-dependent conditions of SAD. Human psychobiotic studies in non-seasonal depressive populations have also yielded non-uniform clinical and mechanistic findings [75,76,77,78]. For LGG specifically, evidence arises from distinct experimental contexts [15,28,80,81,83,84,87], including combined interventions that limit strain-specific attribution [81], while pharmacological evidence involving pathways such as IDO1 [85] does not demonstrate that LGG acts through the same mechanism.

The translational relevance of current experimental models also remains uncertain. Chronic stress- and inflammation-based paradigms commonly used in psychobiotic research do not reproduce the predictable seasonal recurrence, photoperiod dependence, and spontaneous remission characteristic of SAD [80,81]. Photoperiod-based rodent paradigms reproduce selected behavioral and neurobiological responses to altered day length [6,88], but no single model captures the full human phenotype. Differences in species, diurnal or nocturnal activity patterns, light protocols, and behavioral phenotyping further limit cross-study comparison and translation.

Establishing directionality and temporal sequence remains particularly challenging. Seasonal changes in mood, sleep, dietary intake, feeding timing, and physical activity may themselves alter microbial composition and metabolic signaling. A human population study has also reported seasonal variation in gut microbial composition [74], indicating that winter microbiome differences could reflect environmental or behavioral seasonality rather than SAD-specific pathology. Cross-sectional studies are therefore insufficient to determine whether microbiota-associated changes precede, accompany, or follow seasonal symptoms. Repeated within-subject sampling across symptomatic and remission phases, together with assessment of major behavioral and environmental covariates, will be required to improve temporal interpretation.

Mechanistic mediation has also been insufficiently examined. Most studies assess selected components of the proposed framework rather than jointly characterizing circadian timing, barrier and inflammatory measures, microbial function and metabolites, tryptophan–kynurenine metabolism, and neurobehavioral outcomes. Peripheral biomarkers require cautious interpretation because peripheral serotonin does not readily cross the blood–brain barrier, and peripheral tryptophan or kynurenine measures cannot alone establish corresponding changes in central serotonergic function [49,50]. Parallel alterations across biological domains therefore do not demonstrate mediation, and multi-omics associations cannot establish causality without temporal, interventional, or experimentally controlled designs.

Finally, clinical evidence for microbiota-targeted interventions remains heterogeneous across strains, doses, formulations, treatment contexts, dietary backgrounds, baseline microbiota, and clinical phenotypes [56,65,86,89]. Trials in non-seasonal depressive populations have reported non-uniform findings across clinical and mechanistic outcomes [75,76,77,78], limiting generalization between psychobiotic formulations. Direct clinical evidence for LGG in well-characterized SAD populations remains insufficient, and no current evidence demonstrates coordinated regulation of the multiple candidate pathways proposed in this review. Accordingly, the present framework should be interpreted as an evidence map for hypothesis generation and future adjunctive trial design rather than as a validated causal model or demonstration of therapeutic efficacy in SAD.

## 7. Conclusions and Future Perspectives

SAD is increasingly viewed as a multifactorial disorder in which photoperiod-related circadian disturbance is well supported, whereas the contributions of neuroimmune, metabolic, and microbiota-related processes remain less clearly established. Altered feeding rhythms, microbial rhythmicity, and tryptophan metabolism have been proposed as additional factors of potential relevance, but their causal roles and temporal relationships in SAD remain unresolved. LGG provides a mechanistic rationale for future adjunctive investigation, but current evidence derives largely from preclinical or non-seasonal depression contexts and does not establish therapeutic efficacy in SAD.

A major future priority is to adapt photoperiod-relevant experimental paradigms, including short-photoperiod rodent models [88], for evaluating psychobiotic mechanisms under seasonally relevant conditions. Combining such models with longitudinal, multi-omics, and repeated circadian sampling may help clarify temporal relationships, while causal inference will require experimentally controlled perturbations. Experimental research outside SAD suggests that photoperiod can influence gut microbial composition [90]. However, the temporal relevance of these findings to SAD remains unknown, and they should not be interpreted as evidence of an upstream microbiota-driven mechanism.

Direct evidence for microbiota-targeted interventions in well-characterized SAD populations remains limited. Future trials should incorporate seasonally appropriate intervention timing together with circadian, inflammatory, microbiome-related, and tryptophan–kynurenine measures to characterize response heterogeneity and identify biologically relevant subgroups. Dietary composition and meal timing should also be evaluated as potential modifiers of intervention response [69,70,91].

Established evidence-based treatments, particularly bright light therapy, remain the clinical foundation of SAD management. Microbiota-targeted approaches, including LGG, should therefore be evaluated as potential adjuncts rather than alternatives to first-line treatment. Whether combination strategies provide additive or synergistic benefits remains to be established, and future trials may examine whether circadian phenotype, dietary rhythm, baseline microbial composition, inflammatory status, and host metabolic profile predict responses to investigational psychobiotic interventions [91,92,93]. Overall, clinical translation will require demonstration of seasonally relevant mechanisms and reproducible benefit in SAD before precision microbiota-targeted strategies are considered for practice.

## Figures and Tables

**Table 1:** Comparison of established treatments for SAD.

Intervention	Clinical Status	Primary Focus/Mechanism	Key Reported Benefits	Main Limitations	Biological Considerations Not Directly Targeted *
Bright light therapy	Primary treatment with established evidence [4,7]	Promotes circadian phase realignment through timed light exposure and modulation of melatonin timing [4,7,11]	Improves depressive symptoms and sleep-wake timing; may alleviate fatigue [4,7,11]	Variable response; requires adherence and appropriate timing; recurrence may occur after discontinuation [4,7,11,12]	Proposed neuroimmune, metabolic, and microbial processes
SSRIs	Established pharmacological option [9]	Enhances serotonergic neurotransmission by inhibiting serotonin reuptake [9]	Reduces depressive symptom severity in SAD [9]	Variable response; adverse effects and delayed onset may occur; relapse may follow discontinuation [9]	Circadian misalignment and proposed gut microbiota processes
CBT-SAD	Established psychotherapy tailored to SAD [10]	Targets maladaptive seasonal cognitions and behaviors and promotes behavioral activation [10]	Improves coping and seasonal symptom management; may reduce recurrence risk [10]	Requires trained therapists, time commitment, and sustained engagement [10]	Proposed circadian and broader biological processes

* These processes are proposed considerations rather than validated therapeutic targets in SAD.

## Data Availability

No new data were created or analyzed in this study. Data sharing is not applicable to this article.

## Abbreviations

The following abbreviations are used in this manuscript:

SAD	Seasonal affective disorder
5-HT	5-hydroxytryptamine
IDO1	indoleamine 2,3-dioxygenase 1
LGG	*Lacticaseibacillus rhamnosus* GG
HPA	hypothalamic–pituitary–adrenal axis
ipRGCs	Intrinsically photosensitive retinal ganglion cells
SCN	Suprachiasmatic nucleus
NA	Norepinephrine
DA	Dopamine
LPS	Lipopolysaccharide
TPH	Tryptophan hydroxylase
TDO2	tryptophan 2,3-dioxygenase
IL-6	Interleukin-6
TNF-α	Tumor necrosis factor-α
IFN-γ	Interferon-γ
SCFAs	Short-chain fatty acids
AhR	Aryl hydrocarbon receptor
GABA	γ-aminobutyric acid
CBT-SAD	Cognitive behavioral therapy tailored for SAD
SSRIs	Selective serotonin reuptake inhibitors
CUMS	Chronic unpredictable mild stress
MDD	Major depressive disorder
